# Socioeconomic-Related Inequalities in COVID-19 Vulnerability in South Africa

**DOI:** 10.3390/ijerph191710480

**Published:** 2022-08-23

**Authors:** Muna Shifa, David Gordon, Murray Leibbrandt, Mary Zhang

**Affiliations:** 1 Southern Africa Labour and Development Research Unit, University of Cape Town, Cape Town 7700, South Africa; 2 School for Policy Studies, University of Bristol, Bristol BS8 1TH, UK; 3 Oxford School of Global and Area Studies, University of Oxford, Oxford OX2 6LH, UK

**Keywords:** COVID-19, infection prevention, vulnerability index, inequality, concentration index, South Africa

## Abstract

Individuals’ vulnerability to the risk of COVID-19 infection varies due to their health, socioeconomic, and living circumstances, which also affect the effectiveness of implementing non-pharmacological interventions (NPIs). In this study, we analysed socioeconomic-related inequalities in COVID-19 vulnerability using data from the nationally representative South African General Household Survey 2019. We developed a COVID-19 vulnerability index, which includes health and social risk factors for COVID-19 exposure and susceptibility. The concentration curve and concentration index were used to measure socioeconomic-related inequalities in COVID-19 vulnerability. Recentred influence function regression was then utilised to decompose factors that explain the socioeconomic-related inequalities in COVID-19 vulnerability. The concentration index estimates were all negative and highly significant (*p* < 0.01), indicating that vulnerability to COVID-19 was more concentrated among the poor. According to the decomposition analysis, higher income and education significantly (*p* < 0.01) positively impacted lowering socioeconomic-related COVID-19 vulnerability. Living in an urban region, being Black, and old all had significant (*p* < 0.01) positive impacts on increasing socioeconomic-related COVID-19 vulnerability. Our findings contribute to a better understanding of socially defined COVID-19-vulnerable populations in South Africa and the implications for future pandemic preparedness plans.

## 1. Introduction

Non-pharmaceutical interventions (NPIs), such as social distancing, isolation, and regular handwashing, were effective strategies to restrict the spread of COVID-19 before vaccinations were available. Even after introducing a vaccine, such interventions are still necessary, especially in developing countries, where vaccination coverage is relatively low. However, overcrowding, a lack of access to adequate water and sanitation, a lack of medical resources, and pre-existing risk factors (i.e., poor health) plague a large proportion of households and individuals in developing countries [1]. A growing number of studies, mainly from OECD countries, found that the risk of infection and death from COVID-19 was disproportionately higher for people with a disadvantaged socioeconomic status (SES) [2,3,4,5]. Disparities in the capacity to implement NPIs and pre-existing health risk factors are proposed as the primary mechanisms for social inequalities in pandemic morbidity and mortality [6,7]. Due to a lack of data on individual-level COVID-19 infection and mortality, research examining societal disparities in COVID-19 risk factors and NPI compliance is mainly limited to OECD countries.

Using nationally representative survey data from South Africa, this paper aims to fill in this knowledge gap, investigating the extent to which individuals are vulnerable to COVID-19 infection due to their health, socioeconomic, and living circumstances. South Africa is one of the African countries most affected by COVID-19 [8], which is characterised by high SES inequality [9,10]. Previous research in South Africa and elsewhere shows that a lower SES is associated with poor health [11,12,13,14,15] and the inability to practice social distancing [16,17]. For example, based on data from the United States, Papageorge et al. [17] found that people with lower incomes, less outdoor space at home, and fewer opportunities to work from home were less likely to engage in social distancing. Similarly, previous research [16] suggested that social distancing was more common among the relatively rich in South Africa than among the poor. These findings indicate that health and socioeconomic factors influence people’s ability to practice NPIs.

The theory of social determinants of health and the syndemic approach underpin our conceptual framework for assessing COVID-19 vulnerability. In the context of pandemics such as COVID-19, social disparities in pandemic outcomes are caused by differences in virus exposure, susceptibility to the virus, and treatment availability [7,18,19]. The syndemic theory highlights how multiple social, economic, and environmental risks interact and intersect with biological risks, resulting in increased disease risks and health burdens [20,21,22]. According to this theory, pre-existing structural social inequalities cause disparities in the distribution of risk factors and access to health resources, contributing to disparities in health outcomes [22]. Examining disparities in biological and social determinants of health is therefore critical for explaining differences in COVID-19 outcomes and understanding barriers to preventive NPI compliance.

We measured COVID-19 vulnerability using living conditions indicators likely to affect South African families’ ability to implement NPIs and indicators of pre-existing health risk factors. A similar approach has been used in the context of the United States [23]. The few existing studies on COVID-19 vulnerability in South Africa did not investigate SES-related inequalities [1,9]. In addition, unlike previous studies that used a single indicator, such as self-reported social distancing or handwashing, to measure vulnerability to COVID-19, we created an index that includes both health and social risk factors likely to be potential causes of disparities in exposure and susceptibility during pandemics.

Our study aimed to answer two research questions: (1) whether there is an SES gradient in COVID-19 vulnerability, and (2) what factors account for the SES-related inequalities in COVID-19 vulnerability. We used the concentration index (CI) to measure SES-related inequalities in COVID-19 vulnerability. The CI quantifies the degree of association between an individual’s level of COVID-19 vulnerability and their relative position in the income distribution. Recentred influence function (RIF) regression is then used to decompose the factors that explain the SES-related disparities in COVID-19 vulnerability.

Our results show that, even under stringent lockdown policies, low-income households were more likely to be vulnerable to COVID-19 due to their inability to follow NPIs and their pre-existing risk factors. The income gradient on COVID-19 vulnerability revealed that the poor were more likely to be exposed to the virus and susceptible when exposed while bearing the highest economic and social costs of South Africa’s strict lockdown rules [24,25,26].

There are several reasons why people with lower SESs suffer the most severe health consequences during pandemics, even in 21st Century South Africa with its modern social security system and advanced medical care. First, people living in less affluent areas are more likely to catch COVID-19. They are more likely to work in jobs that involve high amounts of social contact (e.g., factory workers, construction workers, care assistants, shop assistants and bus drivers) and, as a result, are more likely to come into contact with infected people than those who may live in more affluent areas and can work from their homes [2,16]. Moreover, individuals in deprived areas are more likely to rely on public transport than those in more affluent areas, thus having more contact with infectious people. Additionally, the higher the population density, the more difficult it is to maintain social distance, and deprived areas tend to have higher population densities than wealthier areas. Therefore, people in deprived areas are more likely to come into contact with infected persons when they leave home for medical care, food shopping, and other activities [1,27].

Second, people with a COVID-19 infection in poor areas are more likely to die. There is a higher risk of severe disease and death from a COVID-19 infection if a person already has underlying health issues, such as hypertension, diabetes, cardiovascular disease, chronic respiratory disease, and cancer [28]. People in deprived areas are more likely to suffer from these health issues than those in more affluent areas for various reasons, including, but not limited to, greater pollution levels, greater stress levels, greater inflammation levels, and greater risk of *Helicobacter pylori* (*H. pylori*) infections in childhood [29]. Unfortunately, the Inverse Care Law reveals that health care quality is often inversely related to health needs, and consequently, on average, deprived areas have worse health care than more affluent areas [30].

## 2. Slowing and Suppressing the Pandemic

Vaccines against SARS-CoV-2 were developed and received regulatory approval for emergency use in fewer than nine months [31]. The global COVID-19 vaccination programme started in the United Kingdom on the 8 December 2020 and began in South Africa on the 17 February 2021. By May 2022, more than 36 million vaccinations had been administered in South Africa, and approximately half of the adult population had received at least one vaccination (see https://sacoronavirus.co.za/latest-vaccine-statistics/ accessed on 30 May 2022).

**Figure 1:**
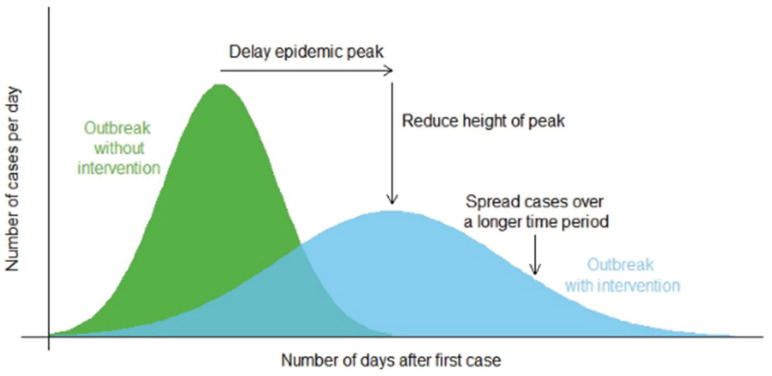
NPIs aim to reduce the rate of infection. Source: World Health Organization [33] (License: CC BY-NC-SA 3.0 IGO).

This still leaves a large percentage of the South African population unvaccinated, and NPIs remain one of the most effective ways to prevent community transmission [32]. Common NPIs include social distancing, facemask wearing, increased ventilation, frequent handwashing with soap and water, etc. The primary purpose of NPIs is to reduce and mitigate the impact of the pandemic by slowing “the spread of infections in the community, delaying the peak in infections, reducing the size of the peak and spreading infections over a longer period of time”, as illustrated in Figure 1 ([33], p. 1). This flattening-the-curve strategy aims to prevent the country’s health service from being overwhelmed by an exponentially increasing number of patients and provide more time for the population to be vaccinated and new treatments to be developed [34].

The purpose of NPIs is to reduce the rate at which a disease spreads (i.e., the disease effective reproduction number “*R*”), defined as the average number of secondary cases per infectious case in a population made up of both susceptible and non-susceptible people [35]. SARS-CoV-2 is a novel virus; therefore, it is assumed that everyone is initially susceptible to contracting a COVID-19 infection [28]. People only gain immunity by being vaccinated or contracting and recovering from COVID-19.

If the effective reproduction number, *R*, is less than 1, the number of infected people will fall; if *R* is 1, the number of people with COVID-19 will stay static, and if *R* is larger than 1, the number of infections will increase [36]. The reproduction number equals the rate of infection divided by the recovery rate (i.e., *R* = Infection rate/Recovery rate). Thus, it can be reduced by either lowering the infection rate (e.g., through social distancing or vaccination) or increasing the recovery rate (e.g., through effective treatments which speed recovery).

The course of a pandemic can be predicted using SEIR (susceptible, exposed, infectious, and recovered) models. In these models, a key parameter is the basic reproduction number (*R*_0_), defined as the average number of people in the susceptible population who will catch the disease from an infected person [37]. However, to implement effective policies for controlling a pandemic, it is crucial to know the average infection rate and “whether specific situations and settings might be driving the outbreak” [38]. For example, in Guangzhou (China), the risk of catching COVID-19 at home (from an infected household member) was approximately ten times greater than the risk of acquiring it in hospital and 100 times greater than the risk on public transport [39,40]. In Japan, contact tracing data showed that the risk of an infected person transmitting the virus was, on average, 18.7 times higher in a closed environment (such as a building or a house) than in an open-air environment [41]. Hence, it is essential for policy purposes to decompose *R*_0_ into a series of Secondary Attack Rates (2^0^AR), i.e., the proportion of people exposed to an infected person who develop the disease within a specific group (e.g., a household or a group of friends). Thereby, adapted according to [37,38], the equation is:

*R*_0_ = 2^0^AR [Household] × Number of contacts [Household] + 2^0^AR [Neighbours/Friends] × Number of contacts [Neighbours/Friends] + 2^0^AR [Community/Strangers] × Number of contacts [Community/Strangers]

Lockdown and social distancing policies mainly reduce contact and the probability of being infected outside the household. However, a quarantine (i.e., not allowing people to leave their homes) is likely to increase the secondary attack rate within households, i.e., where a household member has been infected with the virus before the lockdown [42].

The increased household attack rate may vary by age and the relationship of household members. A meta-analysis of 44 studies by Madewell and colleagues [42] estimated that the average household secondary attack rate of the SARS-CoV-2 variants at that time was 16.6% (95%CI ∈ 14.0–19.3%); the average attack rate for family contacts was 17.4% (95%CI ∈ 12.7–22.5%); and the chance of catching COVID-19 from an infectious household member was significantly higher for adults (28.3%; 95%CI ∈ 20.2–37.1%) than children (16.8%; 95%CI ∈ 12.3–21.7%), with spouses having a much higher risk of a COVID-19 infection (37.8%; 95%CI ∈ 25.8–50.5%). It should be emphasised that serological studies (i.e., studies that measure IgG blood antibodies against COVID-19) have found much higher average household attack rates, e.g., 45% in Norway, 37% in Spain, and 35% in Brazil [43,44,45].

SARS-CoV-2 has evolved to become more infectious. A recent estimate of the average household attack rate for the Delta variant (B.1.617.2), which South Africa first reported at the end of 2021, was 5.1 (i.e., 10 people would, on average, infect another 51 people), whereas that of the original variant of the virus was 2.8 [46]. The average household attack rate for the Omicron variant (B.1.1.52) is about 50% higher than for the Delta variant [47]. Thus, it becomes more critical than ever to understand who in South Africa is at the highest risk of contracting a COVID-19 infection due to their health, socioeconomic, and living conditions and the resulting limited capacity to implement NPIs effectively.

## 3. Data and Methods

### 3.1. Data

The South African General Household Survey (GHS) 2019, a nationally representative sample collected by Statistics South Africa (Stats SA) just before the COVID-19 pandemic, served as the primary data source for this study. The sample was derived using a stratified two-stage sampling method. The first stage sampled primary sampling units (i.e., enumeration areas) using a probability proportional to size method, and the second stage sampled dwelling units (DUs) using systematic sampling [48]. In total, 22,529 DUs were sampled, containing 19,649 households and 68,986 individuals [48]. Individuals with missing values for any of the COVID-19-vulnerability indicators were excluded. After removing these individuals, we had 99.9% (i.e., *n* = 68,924) of the individuals from the full sample. The data included housing conditions, access to essential services, assets, income, health, and demographic factors (i.e., age, gender, and race). In South Africa, race is one of the most important forms of social identification. Statistics South Africa divides the population into four race groups: Blacks/Africans, Coloured, Indians/Asians, and Whites. However, race groups are not homogeneous because they are composed of multiple and diverse ethnic groups.

### 3.2. Measuring COVID-19 Vulnerability

**Figure 2:**
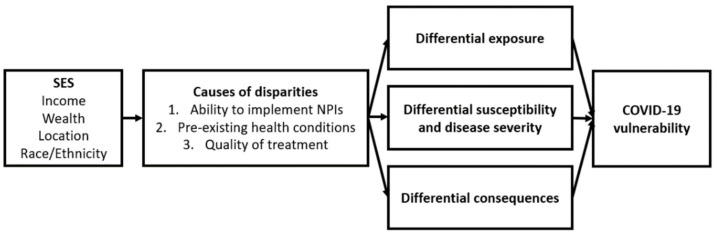
Framework for risk factors leading to unequal COVID-19 vulnerability. Note: Adapted from [7].

Our conceptual framework for assessing disparities in COVID-19 vulnerability in South Africa was based on the theory of social determinants of health and the syndemic approach [7,20,21]. Social disparities in pandemic outcomes are caused by differences in virus exposure, susceptibility when exposed to the virus, and the availability of treatment in the context of pandemics [7,18,19]. The syndemic theory emphasises the interaction and intersection of multiple social, economic, and environmental risks with biological factors, which results in increased disease risks and health burdens [21,22]. Hence, pre-existing health conditions and socioeconomic status can interact with SARS-CoV-2 to determine an individual’s vulnerability to COVID-19 (see Figure 2).

Recent literature reviews on medical and social risk factors during the 1918–1920 influenza pandemic and COVID-19 suggest that the key predictors of pandemic outcomes were chronic diseases, low SES, indigenous status, and location [7]. These factors determine individuals’ health risk factors and the ability to implement preventative measures during pandemics, contributing to disparities in pandemic outcomes. In order to measure people’s vulnerability to COVID-19, we examined an individual’s pre-existing illness and household living conditions that are likely to increase the risk of COVID-19 infection and susceptibility and limit their ability to implement NPIs. Table 1 lists the relevant vulnerability indicators validated by previous scientific findings and applicable in local contexts.

The vulnerability indicators have high face validity resulting from high scientific relevance (i.e., ‘Scientific Reasons and Sources’ in Table 1). These indicators can help identify conditions likely to increase the population’s secondary attack rate. We further examined the vulnerability indicators’ criterion validity by correlating them with aggregated case–fatality data at a district level (see Appendix A for details). We used district level analysis, because there were no data on COVID-19 infections and mortality at the household or individual levels in GHS 2019 or other representative household surveys in South Africa. Except for the toilet-sharing indicator, the findings indicated a statistically significant positive relationship between the vulnerability indicators and the estimated case–fatality rates (i.e., the number of total deaths due to COVID-19 divided by the number of total confirmed COVID-19 cases in each district).

Next, we created an overall vulnerability index based on the nine indicators in Table 1, using two methods to aggregate the nine indicators for each individual to obtain this summary measure of the indicators. First, we calculated a weighted Average Vulnerability Index (AVI) for each person, with each indicator weighted equally. Second, following previous research [9,55], we used a counting approach to aggregate the nine indicators for each individual to assess the Intensity of Vulnerability (IV). We present results based on both methods of aggregation. The AVI ranges from 0 to 1, and the IV scales range from 0 (no vulnerability in any of the indicators) to 9 (vulnerable in all nine indicators). Higher AVI and IV values indicate a higher vulnerability to COVID-19 infection. We used STATA software (version 17, StataCorp LLC, College Station, TX, USA) to analyse the data.

### 3.3. SES-Related Inequalities in COVID-19 Vulnerability

This section discusses the empirical strategies used to analyse SES-related inequalities in COVID-19 vulnerability. First, the relationships between COVID-19 vulnerability and SES and demographic factors were investigated using regression analysis. Second, the concentration curve and the associated concentration index were used to investigate SES-related inequalities in COVID-19 vulnerability. Finally, a recentred influence function decomposition analysis was implemented to identify factors contributing to SES-related inequalities in COVID-19 vulnerability. Each of these is briefly described subsequently.

### 3.4. Regression Model

To investigate the associations between COVID-19 vulnerability and SES (education and income) and demographic factors (age, race, and gender), we specified the following model:

$$Y_{i}=X^{\prime }\beta +\varepsilon_{i}$$

where $Y_{i}$ is individual *i*’s COVID-19 vulnerability score (i.e., AVI and IV, respectively), $X^{\prime }$ is a vector of explanatory variables, *β* is a vector of coefficients of the explanatory variables, and $\varepsilon_{i}$ is the error term, assumed to have a conditional mean of zero.

Ordinary least squares (OLS) regression was applied to estimate the model [56]. Fractional probit regression was also conducted because the AVI ranged from 0 to 1 [56]. Although IV ranged from 0 to 9, only a few people reported as being vulnerable in eight or nine indicators. Hence, scores 8 and 9 were combined with 7 for the regression analyses.

### 3.5. The Concentration Curve and Concentration Index

We estimated SES-related inequalities in COVID-19 vulnerability using the concentration curve and the concentration index, which are widely adopted to measure SES-related health inequalities [57,58,59,60]. The concentration curve plots the cumulative percentage of a health variable on the vertical axis against the cumulative share of the population ranked by SES (from lowest to highest) on the horizontal axis. A concentration curve above the line of equality (i.e., the 45-degree line) indicates greater COVID-19 vulnerability amongst the poor, whereas a concentration curve below the line of equality indicates greater vulnerability amongst the rich. If everyone had the same level of vulnerability, regardless of SES, the concentration curve would overlap with the 45-degree line. The greater the degree of inequality, the further the concentration curve diverges from the line of equality.

The associated concentration index (CI) is a summary index, defined as twice the covariance between a health variable and an SES ranking [58]:

$$\mathrm{CI}=\frac{2}{\mu_{h}}cov\left(Y_{h},R\right)$$

where $Y_{h}$ is the individual COVID-19 vulnerability index score (i.e., AVI and IV), $\mu_{h}$ represents the mean of the index, and *R* represents the socioeconomic ranking of the individual. The CI quantifies the degree of association between an individual’s level of COVID-19 vulnerability and their relative position in the socioeconomic distribution. For our socioeconomic ranking, we used the per capita household income.

AVI and IV are both bounded variables; thus, the standard CI has limitations. For bounded health measures, for example, the magnitude of the CI differs when calculated using the good health indicator versus when calculated using the associated ill health indicator [61]. In addition, the minimum and maximum values of the CI are dependent on the mean of the health indicator [62]. To account for these specific issues, Erreygers [61] and Wagstaff [62] each proposed a “corrected” CI for use when the health measure was bounded [58]. However, there is no agreement on which of the two normalisations is preferable. According to Erreygers and Van Ourti [58], Erreygers’ index [61] has some desirable properties compared with Wagstaff’s [62] index. We thus used the Erreygers’ [61] normalised concentration index (EI). This is calculated according to:

$$\mathrm{EI}=\frac{4\mu_{h}}{b_{h}-a_{h}}\times \frac{2}{\mu_{h}}cov\left(Y_{h},R\right)$$

The CI ranges from −1 to +1, with a negative value indicating that COVID-19 vulnerability is disproportionately concentrated among the poor and a positive value indicating that COVID-19 vulnerability is disproportionately concentrated among the rich. The index assigns a value of zero if there is an absence of SES-related inequalities in COVID-19 vulnerability.

### 3.6. Decomposing the Concentration Index of COVID-19 Vulnerability

Recentred influence function (RIF) regression was used to decompose the concentration indices of COVID-19 vulnerability. RIF regression is recommended for decomposing bivariate rank-dependent indices such as the CI to identify factors contributing to SES-related health inequalities [63]. It overcomes the main limitation of the most commonly used alternative decomposition methods, such as the Wagstaff decomposition method, which only explains the degree of variation in health while ignoring the covariance between SES rank and health [63].

The RIF method uses standard regression techniques to estimate the unconditional partial effects of small changes in a given explanatory variable on the size of distributional statistics such as a CI [63,64,65]. The procedure involves two steps. First, each observation’s RIF value, i.e., a transformation of the influence function (IF), is computed. The IF quantifies the influence of a given observation on the estimation of the CI, whereas the RIF quantifies the relative contribution of that observation to the CI [65]. The average of individual RIF is the CI itself.

Under the assumption of a linear relationship between the RIF and the explanatory variables and an additive error term with a conditional zero mean value, the RIF regression can be estimated using the OLS approach, which is termed RIF-I-OLS regression. Rios-Avila [65] provides a simple procedure to estimate RIF-regression using STATA software, with the formula for RIF-I-OLS given as [65]:

$$\mathrm{RIF}\left\{h,v\left(F_{H\;}\right)\right\}=X^{\prime }\beta +\varepsilon_{i}$$

where h is the COVID-19 vulnerability index, $v\left(F_{H}\right)$ is the distribution statistic (CI of COVID-19 vulnerability), X is the same vector of explanatory variables that were used in the OLS regression, and $\varepsilon_{i}$ is an error term assumed to have a zero conditional mean. The coefficient β is the marginal effect of the explanatory variables on the RIF of the CI.

## 4. Results and Discussion

### 4.1. Distribution of COVID-19 Vulnerability

**Figure 3:**
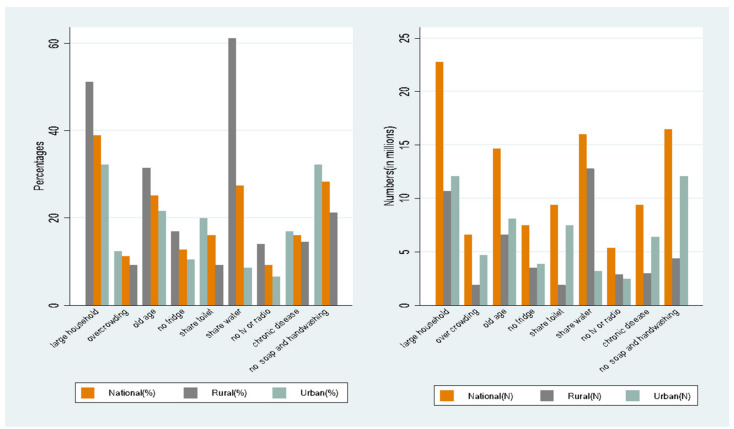
Vulnerability indicators, nationally and in rural and urban areas. Source: Authors’ calculation using GHS 2019.

Figure 3 shows the differences between our vulnerability indicators at national, rural, and urban levels. There were remarkable differences between rural and urban areas regarding the extent of lacking on-site water, household size, and the presence of older people. Approximately 62% of people (16 million) in rural areas lived in households that lacked on-site water sources, whereas the figure in urban areas was only 8.6% (3.2 million individuals). By contrast, approximately 20% of people (7.5 million) in urban areas lived in households where a toilet was shared with another household, whereas only 9.3% of people in rural areas (1.9 million) lived in similar conditions. Approximately 14% of households in rural areas lacked access to television or radio, whereas only 7% of the households in urban areas were estimated to be without access. The proportion of the population without access to soap and handwashing facilities was higher in urban (32%) than in rural areas (21%).

A set of descriptive statistics for the two vulnerability indices (i.e., AVI and IV) by demographic and SES factors is shown in Table 2. Individuals from lower-income households were more vulnerable than those from higher-income households. Those in the lowest income quintile had an AVI of 0.26 compared with 0.12 for those in the highest quintile. Members of female-headed households were more vulnerable than those of male-headed households. Similarly, older people were more likely to live in households with a limited capacity to comply with NPIs and had pre-existing health conditions, contributing to their vulnerability. These findings were consistent with previous research, indicating a link between older age and female-headed households and poverty in South Africa [66,67].

### 4.2. Inequalities in COVID-19 Vulnerability

Table 3 displays the regression analyses in which AVI and IV were dependent variables. An increased risk of COVID-19 vulnerability was associated with having a lower level of education and income and being African/Black. There was no significant difference between having a primary education and not having any education. On the other hand, having a secondary or tertiary education was significantly associated with a lower risk of COVID-19 vulnerability. These findings suggest that people with a lower SES were more vulnerable to COVID-19 than those with higher SES. After controlling for income, the significant impact of education remains, suggesting that, given equal income levels, more educated individuals may have better access to information and opportunities for living in better housing conditions (e.g., less crowding and with access to water and sanitation). They were also more likely to be in better health because of improved health literacy and associated health-promoting behaviour [68].

**Figure 4:**
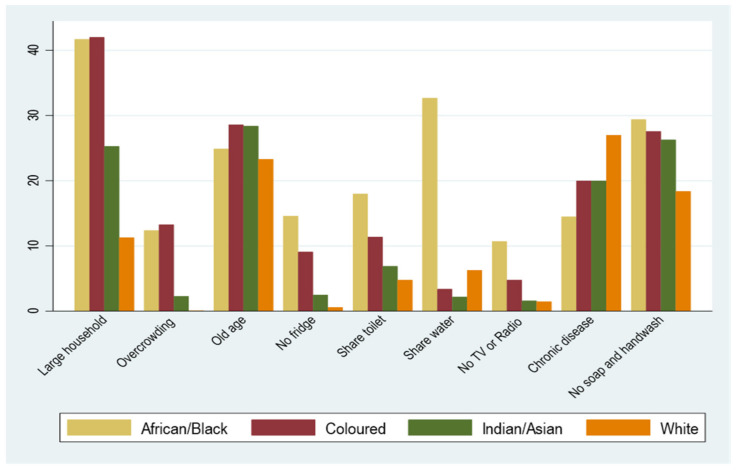
COVID-19 vulnerability indicators by race in percentages. Sources: Authors’ calculation using GHS 2019.

The significance of race was as expected, given that inequality in South Africa continues to have a substantial racial dimension, including in defining residential areas due to the country’s history of segregation regulations [10]. Thus, given the same income and education levels, Blacks remained more likely to be vulnerable to COVID-19 than other race groups. They were vulnerable in most vulnerability indicators (see Figure 4). Compared with Whites, Africans/Blacks had a much larger proportion of people who shared water and toilet facilities, lived in large and overcrowded households, and did not have access to a refrigerator, information (TV or radio), or handwashing facilities.

In contrast to the descriptive results, the gender of the head of household became statistically insignificant once income was controlled. This is probably due to the strong correlation between low income and female headship. Due to their living conditions, individuals living in rural areas were more vulnerable to COVID-19 than those in urban areas. This is probably partially because rural populations have larger family sizes and are more likely to share water facilities than urban residents. Overall, our findings imply that people with higher SESs were more likely to live in households that can comply with NPIs and less likely to have pre-existing health conditions than people with lower SESs, making them less vulnerable to COVID-19. Such findings are consistent with previous research which shows that people from disadvantaged backgrounds were less able to implement preventive measures such as social distancing [16,17] during pandemics and were more likely to have higher susceptibility due to the presence of pre-existing health risk factors [7,69].

**Figure 5:**
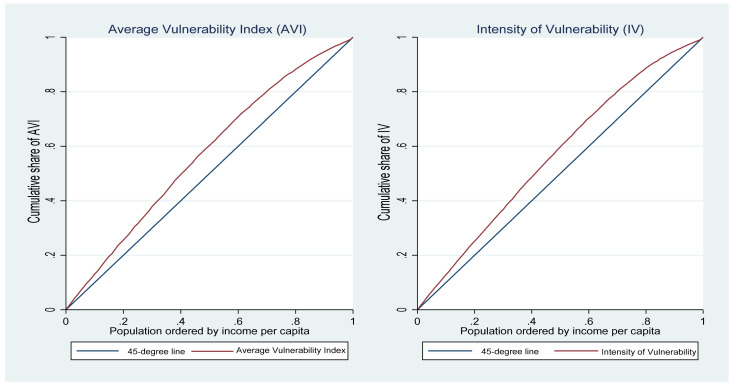
Concentration curves for COVID-19 vulnerability. Source: Authors’ calculation using GHS 2019.

Figure 5 depicts the concentration curves for the AVI and IV measures. The concentration curves for both measures were above the 45-degree line, suggesting that those with low per capita household income were disproportionately vulnerable to COVID-19 infection.

We also used a wealth index as an alternative SES ranking variable to per capita household income as a robustness check. The wealth concentration curves were similar to the per capita household income ranking. We used the uncentered PCA (UCPCA) method to create our wealth index from a set of household asset indicators [70]. The asset set included a stove, a vacuum cleaner, washing machines, a phone, a table, a personal computer, a satellite dish, a car, a DVD player, a home theatre, a microwave, a geyser, and an air conditioning system. Table 4 shows the estimates of the CI using income (columns 1 and 2) and a wealth index (columns 3 and 4) as SES ranking variables. The CI estimates were all negative and highly significant (*p* < 0.01). This indicates that vulnerability to COVID-19 was more concentrated among the poor, which is consistent with the concentration curve analysis. It again indicates that the rich, compared with the poor, can afford to live in households that allow them to implement NPIs and have better health conditions, potentially due to the social determinants of health and their access to better healthcare [71].

Table 5 shows the outcome of the concentration index decomposition based on RIF-I-OLS regression. Except for gender, all factors significantly impacted inequality in SES-related COVID-19 vulnerability. Higher income reduced inequalities in SES-related COVID-19 vulnerability because increased income improves living conditions. Completing primary or secondary education was associated with increased SES-related COVID-19 vulnerability inequality, whereas tertiary education significantly reduces SES-related COVID-19 vulnerability inequality. In other words, increasing the percentage of people with a tertiary education can reduce income-related inequalities in COVID-19, probably because tertiary education is associated with higher earnings in South Africa than lower educational attainment levels [72], and graduates are also likely to be better off in other dimensions of wellbeing.

Individuals from other races were more likely than Africans/Blacks to be negatively associated with SES-related COVID-19 vulnerability inequalities. This could be because, compared with other race groups, Africans/Blacks are more likely to live in areas with poor housing conditions holding income constant [73], thereby increasing inequalities in SES-related COVID-19 vulnerability between the rich and the poor. Older age significantly positively impacted inequalities in SES-related COVID-19 vulnerability. Similarly, living in urban areas significantly positively impacted inequalities in COVID-19 vulnerability compared with living in rural areas. As a result, increasing the proportion of people living in urban areas increases inequalities in SES-related COVID-19 vulnerability.

## 5. Discussion

A growing body of research suggests that disparities in health and social risk factors may contribute to COVID-19 outcome disparities across social groups. In this study, we empirically examined SES-related inequalities in COVID-19 vulnerability using nationally representative survey data from South Africa. Examining social disparities in exposure and the consequences of pandemic outcomes is critical for implementing policies that consider pre-existing social inequities when coping with pandemics.

Our findings show disparities in exposure risk and susceptibility across social groups, reflecting structural inequalities in South Africa. When comparing the distribution of COVID-19 vulnerability across social groups, we found that being African/Black, living in rural areas, and being older were associated with an increased risk of COVID-19 vulnerability. By contrast, higher income and education were associated with a lower risk of COVID-19 vulnerability. After controlling for other factors, we found no significant relationship between the gender of the household head and COVID-19 vulnerability.

The negative and significant association between COVID-19 vulnerability and higher income and education level suggests that individuals with higher income and education can afford to live in areas with better housing conditions [73], allowing them to implement NPIs. Additionally, they are less likely to have pre-existing health conditions [11,12,13,14], reducing their COVID-19 exposure and susceptibility. A similar argument can be made for the positive and significant association between being Black and having a higher COVID-19 vulnerability. In South Africa, a large proportion of Blacks, compared with other race groups, live in areas with poor housing conditions [73] and report poor health outcomes [13]. These findings are consistent with previous research, showing that people with low SES and disadvantaged backgrounds were less able to implement NPIs such as social distancing [16,17] during pandemics and were more likely to be susceptible due to the presence of pre-existing health risk factors [7,69].

Based on our analysis of the SES gradient of COVID-19 vulnerability, we found significant and negative CI coefficient estimates, indicating that COVID-19 vulnerability is more concentrated among the poor in South Africa (i.e., pro-poor). Those with a lower SES are more likely to live in areas with poor housing conditions and are more likely to have poor health conditions.

Examining the factors contributing to SES-related inequalities in COVID-19 vulnerability revealed that higher income and tertiary education negatively and significantly influenced SES-related COVID-19 vulnerability. Individuals with higher income and education were more likely to have a higher SES ranking (i.e., income or assets) and less vulnerability to COVID-19. Thus, increasing the proportion of individuals with higher income and education is associated with a decrease in SES-related COVID-19 vulnerability. Compared with other race groups, being African/Black was associated with increased SES-related COVID-19 vulnerability. This group had a relatively lower SES ranking than other race groups and was more vulnerable to COVID-19. This is consistent with South Africa’s long-term racial inequalities. For example, the annual median income among Whites is eleven times greater than that of Blacks, whereas the median income among Indians is four times greater than that of Blacks [73].

Similarly, compared with other race groups, a significantly higher proportion of Africans/Blacks had to share water and toilet, lived in large and overcrowded households, and did not have access to a refrigerator, information (TV or radio), or handwashing facilities. Furthermore, Blacks experience higher within-group income and asset inequalities than Whites [73]. A greater disparity between Blacks and Non-Blacks and higher within-Black disparities may contribute to the increased SES-related COVID-19 vulnerability.

The regression analysis on AVI and VI (i.e., Table 3) showed a significant negative urban (vs. rural) coefficient, whereas the RIF-I-OLS regression (i.e., Table 5) presented a significant positive coefficient on the urban (vs. rural) comparison. This is because the former explored the expected value of vulnerability among rural and urban residents, whereas the latter examined the contribution that each factor can explain inequalities. One explanation for this finding is that, on average, inequality is greater in urban areas of South Africa than in rural areas [73], and urban areas have higher multidimensional inequalities than rural areas. The higher within-urban economic inequality resulted in higher SES-related inequality in COVID-19 vulnerability. Thus, reducing SES-related inequities in COVID-19 vulnerability necessitates reducing both rural–urban inequalities and inequalities within urban areas. However, it is also important to note that the COVID-19 vulnerability gap between urban and rural areas was not as large as the gap between racial groups (see Figure 3).

The SES-related disparities in exposure and susceptibility during pandemics such as COVID-19 have significant policy implications. The role of race and SES in predicting COVID-19 vulnerability can be attributed to continued inequalities in access to essential services and living conditions, which undermine the implementation of NPIs to reduce COVID-19 spread in disadvantaged communities. Thus, vaccine access should not only consider medical risk factors, but also social risk factors. Access to vaccinations for people living in areas with poor infrastructure, such as access to water and sanitation and densely populated areas, becomes crucial in limiting the virus’s spread and impact. Effective and targeted communication about COVID-19 and vaccinations is also critical in promoting vaccination and countering vaccination reluctance in such communities.

Socially disadvantaged groups are more likely to be vulnerable to COVID-19 risk. Despite strict lockdown rules, there are disparities in the ability to limit exposure to the virus based on SES. This means that not only will the poor bear a greater economic burden, such as job loss, due to South Africa’s strict lockdown policies [24,25,26,74], but they also have limited support to reduce their exposure and susceptibility during pandemics.

Our COVID-19 vulnerability analysis is based on probable factors that cause disparities in exposure and susceptibility to COVID-19 risk. We created a composite measure to account for both health and social factors that influence COVID-19 exposure and susceptibility once exposed. This study, however, had limitations. First, we did not have data on actual COVID-19 incidences or complications at the individual level or disaggregated by social factors to compare our COVID-19 vulnerability index. Second, we could not include access to quality medical treatments due to a lack of data, which has implications for the differences in the consequences of exposure to the virus. With more data on additional factors such as the quality of medical treatments available, future research can examine how well the vulnerability assessments in this study and other comparable studies predict inequalities in actual COVID-19 outcomes. Research of a similar nature, therefore, is needed.

## 6. Conclusions

Understanding socioeconomic disparities in pandemic outcomes due to health and social risk factors is critical for pandemic preparedness strategies [7]. In this study, we found that individuals with lower SESs were more vulnerable to COVID-19 due to their pre-existing risk factors and limited capacities to follow NPIs. The income gradient on COVID-19 vulnerability suggests that the poor were more likely to be exposed to the virus and more likely to be susceptible when exposed. As part of its future pandemic preparedness programme, the South African government must take rapid steps to improve the poor’s health and living standards to reduce SES-related inequities in pandemic outcomes.

## Figures and Tables

**Table 1:** COVID-19 vulnerability indicators.

Vulnerability Indicators	Secondary Attack Rate Level	Scientific Reasons and Sources
Large household with six or more people.	Household	An ill person is more likely to infect their household members than friends, neighbours, or the wider community. The larger the household, the more members are likely to be infected [49].
People over 60 live in households with one or more younger people aged between 7 and 60 years.	Household	People aged 60 and over are more likely to die or suffer from a severe COVID-19 infection. Older people are more likely to be infected within households with younger members, i.e., older people have a higher secondary attack rate within the household [50].
Overcrowded household with more than three people per room.	Household	COVID-19 is primarily spread by contact with coughed and respired droplets and fomites. It is difficult or impossible for household members to socially distance themselves from an infected household member in overcrowded households [5].
No refrigerator.	Household	Those living in households without a refrigerator need to leave their homes more frequently to acquire food; thus, they are at greater risk of infection [51].
No access to handwashing facilities and lack of soap for handwashing.	Household	Inability to wash hands regularly with soap or detergent increases the risk of contracting COVID-19 [52].
A household member with a chronic health condition.	Household	Individuals with chronic health conditions are more likely to suffer from a more severe COVID-19 infection and remain infectious for longer [50].
No access to a radio or TV.	Household	Effective risk communication and community engagement are crucial to controlling infectious disease epidemics. It is difficult for households without access to broadcast media to source the correct public health information to stay safe, because misinformation and rumour during a pandemic can be extensive and dangerous [53].
Sharing a toilet with other households or not having a toilet facility.	Wider community	Sharing a toilet increases the risk of catching COVID-19 from infected people in neighbours’ households either by faecal/oral transmission or from close contact in or near the shared toilet, e.g., while waiting or queuing [54].
Water source not in house or yard/plot of dwelling.	Wider community	Collecting water from a public supply increases the risk of catching COVID-19 from infected people in other households due to close contact while queuing to collect water or touching contaminated water supply equipment, e.g., stand-pipe taps, pump handles, well buckets, etc. [54].

**Table 2:** Vulnerability indices by demographic and socioeconomic factors.

	AVI (0–1)	IV (0–9)
Old (age > 60 years)		
No	0.20	1.82
Yes	0.27	2.41
Head gender		
Male	0.19	1.72
Female	0.23	2.04
Income		
Quintile 1	0.26	2.33
Quintile 2	0.24	2.18
Quintile 3	0.22	2.00
Quintile 4	0.19	1.70
Quintile 5	0.12	1.04
Race		
African/Black	0.22	1.99
Coloured	0.18	1.60
Indian/Asian	0.13	1.15
White	0.10	0.93
Education		
No Education	0.23	2.09
Primary	0.24	2.14
High School	0.20	1.79
Tertiary	0.13	1.15
Region		
Urban	0.18	1.61
Rural	0.25	2.29

Source: Author’s calculation using GHS 2019.

**Table 3:** Regression of COVID-19 vulnerability indices.

	(1)	(2)	(3)
	AVI (OLS)	AVI (Fractional Regression)	VI (OLS)
Old (age > 60 years)	0.07 ***	0.25 ***	0.66 ***
	(0.00)	(0.00)	(0.00)
Female	0.00	0.01	0.02
	(0.39)	(0.37)	(0.40)
Reference = African/Black			
Coloured	−0.03 ***	−0.11 ***	−0.26 ***
	(0.00)	(0.00)	(0.00)
Indian/Asian	−0.04 ***	−0.21 ***	−0.39 ***
	(0.00)	(0.00)	(0.00)
White	−0.06 ***	−0.28 ***	−0.52 ***
	(0.00)	(0.00)	(0.00)
Log pc income	−0.03 ***	−0.10 ***	−0.25 ***
	(0.00)	(0.00)	(0.00)
Reference = No education			
Primary	0.00	0.01	0.01
	(0.42)	(0.31)	(0.41)
Secondary	−0.02 ***	−0.05 ***	−0.14 ***
	(0.00)	(0.00)	(0.00)
Tertiary	−0.04 ***	−0.16 ***	−0.34 ***
	(0.00)	(0.00)	(0.00)
Urban	−0.05 ***	−0.18 ***	−0.48 ***
	(0.00)	(0.00)	(0.00)
Constant	0.46 ***	0.08 *	4.14 ***
	(0.00)	(0.02)	(0.00)
R-squared/Pseudo R^2^	0.17	0.02	0.17
N	67,733	67,733	67,733

Source: Author’s calculation using GHS 2019. Notes: Columns (1) and (3) show *b*-coefficient estimates of the AVI and IV dependent variables using the OLS regression approach, whereas column (2) shows the estimates of AVI using the fractional regression method; all models included province fixed effects; * *p* < 0.05, ** *p* < 0.01, *** *p* < 0.001.

**Table 4:** Concertation index of SES-related vulnerability to COVID-19.

	(1)	(2)	(3)	(4)
	Income-Ranking		Wealth-Ranking	
	AVI	IV	AVI	IV
EI	−0.118 ***	−0.152 ***	−0.149 ***	−0.192 ***
	(0.0037)	(0.0048)	(0.00367)	(0.0046)
N	68,254	68,254	68,923	68,923

Source: Authors’ calculation using GHS 2019. Notes: Columns (1) to (4) show CI estimates of the AVI and IV variables; standard errors are in parentheses; * *p* < 0.05, ** *p* < 0.01, *** *p* < 0.001.

**Table 5:** RIF-I-OLS regression for COVID-19 vulnerability indices.

	(1) EI (AVI)	(2) EI (IV)
Old (age > 60 years)	0.03 ***	0.31 ***
	(0.00)	(0.00)
Female	0.01	0.10
	(0.05)	(0.05)
Reference = African/Black		
Coloured	−0.03 **	−0.27 **
	(0.00)	(0.00)
Indian/Asia	−0.12 ***	−1.10 ***
	(0.00)	(0.00)
White	−0.18 ***	−1.61 ***
	(0.00)	(0.00)
Log pc income	−0.02 ***	−0.21 ***
	(0.00)	(0.00)
Reference = No education		
Primary	0.01	0.07
	(0.05)	(0.05)
Secondary	0.03 ***	0.28 ***
	(0.00)	(0.00)
Tertiary	−0.04 ***	−0.35 ***
	(0.00)	(0.00)
Urban	0.03 ***	0.23 ***
	(0.00)	(0.00)
Constant	0.04	0.39
	(0.13)	(0.13)
R-squared	0.05	0.05
N	67,733	67,733

Source: Author’s calculation using GHS 2019. Notes: Columns (1) and (2) show *b*-coefficient estimates from the RIF-I-OLS decomposition; all models included province fixed effects; * *p* < 0.05, ** *p* < 0.01, *** *p* < 0.001.

**Figure A1:**
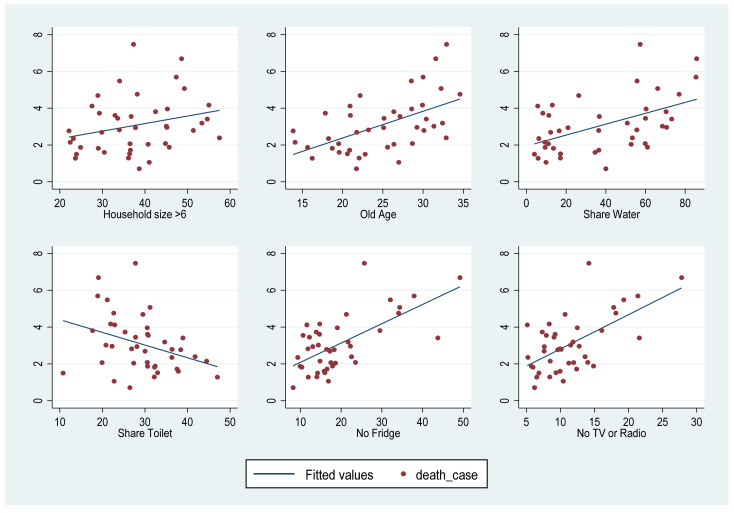
Deaths/cases vs. COVID-19 vulnerability indicators (by district). Source: Authors’ calculation using data from the CS 2016 and provincial Health Department websites.

## Data Availability

The data were stored and managed by DataFirst of the University of Cape Town via https://www.datafirst.uct.ac.za/dataportal/index.php/catalog/central accessed on 17 February 2021.

## Appendix A

The vulnerability indicators used in this paper have high face validity due to their high scientific relevance (see Table 1). It is also important to test the indicators’ criterion validity to determine how closely they are related to the risk of COVID-19 infections and mortality. Criterion validity analysis requires information on COVID-19 infection and mortality at the household or individual level and SES. Such data are difficult to obtain in countries such as South Africa. COVID-19 infection and mortality data are also scarce at lower geographical levels. Therefore, we could only obtain data on cumulative COVID-19 cases and deaths at the district level for seven of the nine provinces in South Africa. This information was analysed to evaluate the criterion validity of some of the COVID-19 vulnerability indicators used in this paper. Our analysis included 41 districts out of the country’s 52 districts. Western Cape and Free State were two provinces with no available data.

Given that our sample from the 2019 GHS was not representative at the district level, we used data from the 2016 community survey (CS) to measure COVID-19 vulnerability based on similar indicators used in the 2019 GHS. However, the 2016 CS only included six of the nine indicators used to assess COVID-19 vulnerability. Thus, our criterion validity test was based on the six vulnerability indicators from the 2016 CS. Shifa et al. [7] demonstrated that measuring COVID-19 vulnerability using indicators from the 2016 CS and the 2018 GHS produced similar results. Hence, we did not anticipate a significant difference in COVID-19 vulnerability measures based on the 2016 CS and the 2019 GHS.

Given that reported cases and death numbers are a function of testing rates and practices that vary over time and space, we considered using the case–fatality rate for criterion validity analysis. The total number of COVID-19 cases and a total number of COVID-19 deaths reported by each province between 16th and 22nd January 2022 were used to calculate the case–fatality rates. Figure A1 depicts the district-level relationship between the case–fatality rates and vulnerability indicators from the 2016 CS. Except for the toilet sharing indicator, the findings showed a statistically significant positive relationship between the vulnerability indicators and the COVID-19 case–fatality rates. Further research should be conducted to understand why the expected positive linear relationship was not found for the toilet sharing indicator.
